# Newly Developed Sarcopenia as a Prognostic Factor for Survival in Patients who Underwent Liver Transplantation

**DOI:** 10.1371/journal.pone.0143966

**Published:** 2015-11-30

**Authors:** Ja Young Jeon, Hee-Jung Wang, So Young Ock, Weiguang Xu, Jung-Dong Lee, Jei Hee Lee, Hae Jin Kim, Dae Jung Kim, Kwan Woo Lee, Seung Jin Han

**Affiliations:** 1 Department of Endocrinology and Metabolism, Ajou University School of Medicine, Suwon, Republic of Korea; 2 Department of Surgery, Ajou University School of Medicine, Suwon, Republic of Korea; 3 Office of Biostatistics, Ajou University School of Medicine, Suwon, Republic of Korea; 4 Department of Diagnostic Radiology, Ajou University School of Medicine, Suwon, Republic of Korea; University of Modena & Reggio Emilia, ITALY

## Abstract

**Introduction:**

The relationship between a perioperative change in sarcopenic status and clinical outcome of liver transplantation (LT) is unknown. We investigated whether post-LT sarcopenia and changes in sarcopenic status were associated with the survival of patients.

**Method:**

This retrospective study was based on a cohort of 145 patients from a single transplant center who during a mean of 1 year after LT underwent computed tomography imaging evaluation. The cross-sectional area of the psoas muscle of LT patients was compared with that of age- and sex-matched healthy individuals. The Cox proportional hazards regression model was used to determine whether post-LT sarcopenia and changes in sarcopenic status affect post-LT survival.

**Results:**

The mean age at LT of the 116 male and 29 female patients was 50.2 ± 7.9 years; the mean follow-up duration was 51.6 ± 32.9 months. All pre-LT patients with sarcopenia still had sarcopenia 1 year after LT; 14 (15%) patients had newly developed sarcopenia. The mean survival duration was 91.8 ± 4.2 months for non-sarcopenic patients and 80.0 ± 5.2 months for sarcopenic patients (log-rank test, p = 0.069). In subgroup analysis, newly developed sarcopenia was an independent negative predictor for post-LT survival (hazard ratio: 10.53, 95% confidence interval: 1.37–80.93, p = 0.024).

**Conclusion:**

Sarcopenia in LT recipients did not improve in any of the previously sarcopenic patients and newly developed within 1 year in others. Newly developed sarcopenia was associated with increased mortality. Newly developed sarcopenia can be used to stratify patients with regard to the risk of post-LT mortality.

## Introduction

Liver transplantation (LT) is a definitive treatment for patients with end-stage liver disease, acute liver failure, and primary hepatic malignancy [1]. Among the many factors that contribute to survival and outcome after LT are donor- and recipient-related variables, immunosuppressive therapy management, and surgical factors. Marked advances in surgical technique, organ preservation, perioperative management, and immunosuppression as well as improved donor and recipient selection have increased the survival rates of LT recipients, and survival beyond the critical period of 6 months after LT has shown a steady increasing trend since 2000 [2, 3]. However, the factors that influence the long-term survival of patients are poorly understood.

Sarcopenia, defined as a loss of skeletal muscle mass and muscle function [4, 5], is more prevalent in patients with cirrhosis than in the general population and is related to adverse clinical outcomes [6–8]. The relationship between sarcopenia and LT outcomes has been examined in several recent studies. Pre-LT sarcopenia is associated with elevated postoperative LT complications and longer hospital stay [9–13] and is a predictor of mortality following LT [13–16]. However, whether pre-LT sarcopenia is negatively associated with long-term survival after LT is controversial [10, 11]. Furthermore, the relationship between a perioperative change in sarcopenic status or of post-LT sarcopenia and clinical outcome has yet to be determined. In a study by Tsien et al [17], patients with loss of muscle mass post-LT showed a trend toward a higher mortality, but due to the small number of events and the small size of the study population, this association did not reach statistical significance.

In this study, we investigated whether post-LT sarcopenia and changes in sarcopenic status, as assessed by computed tomography (CT) scans performed just before LT and at a mean of 1 year after LT, were associated with survival of patients after the critical 6-month period.

## Patients and Methods

### Ethics Statement

This study was approved by the institutional board of Ajou University Hospital and confirmed to the ethical guidelines of the Declaration of Helsinki. Patient records and information were anonymized and de-identified prior to analysis. None of the transplant donors were from a vulnerable population and all donors or next of kin provided written informed consent that was freely given.

### Study Participants

**Fig 1:**
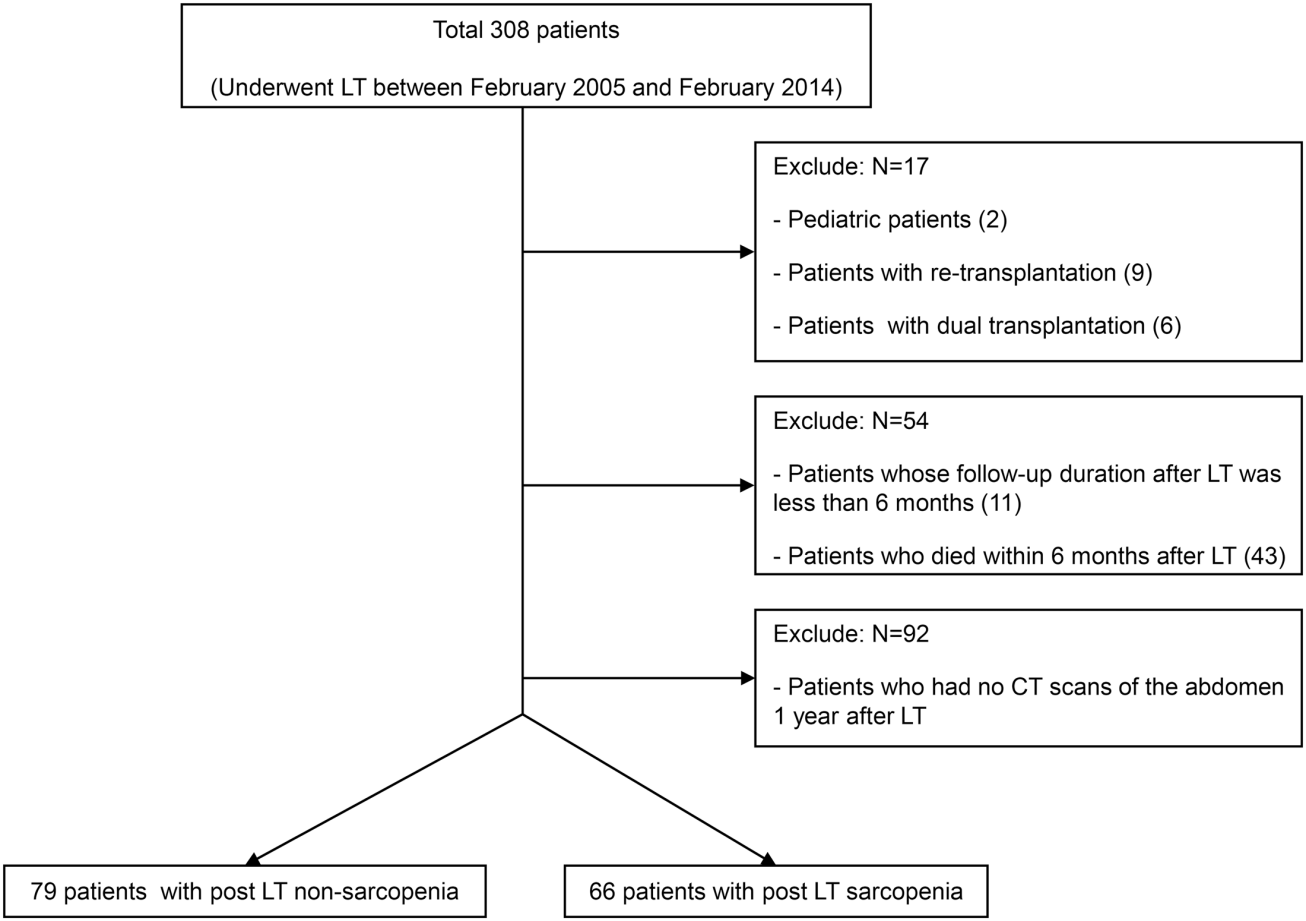
Study population framework. LT, liver transplantation, CT, computed tomography

This study was a retrospective study based on a cohort of patients who underwent LT at Ajou University Hospital (Suwon, Republic of Korea). The characteristics of these patients were reported previously [18–20]. Between February 2005 and February 2014, 308 patients underwent LT. The present study included 145 adult LT patients who had undergone abdominal CT scans just before and 1 year after LT (Fig 1). Pediatric patients (n = 2), patients with re-transplantation (n = 9) or dual transplantation (n = 6), patients whose follow-up duration after LT was less than 6 months (n = 11), patients who died within 6 months after LT (n = 43), and patients (n = 92) who had no abdominal CT scans 1 year after LT were excluded from the study. Only LT recipients who also underwent a postoperative CT scan at a mean of 1 year after LT were included, such that our results may not be applicable to all LT recipients. However, the clinical characteristics of the excluded patients who did not have postoperative CT scans, such as age (49.2 ± 9.0 years), BMI (24.0 ± 4.0 kg/m^2^), sex (71.7% males), and donor age (35.1 ± 14.5 years) were not significantly different from those of the final study population. Postoperative mortality as an outcome of the non-study patients group without a postoperative CT was not significantly different compared to the study patients group (adjusted HR, 0.90; 95% confidence interval, 0.37–2.15; p = 0.803). None of the patients underwent any pretransplant sarcopenic intervention, such as nutrition, physical therapy or a modification of their medications.

### Clinical and Laboratory Assessment

Medical records were used to collect all relevant data. Recipient-related factors included sex, age, body mass index (BMI), Child–Pugh and model for end-stage liver disease scores, presence of hepatic encephalopathy, fasting plasma glucose, diabetes mellitus (DM), pathology necessitating LT, presence of hepatocellular carcinoma (HCC), hypertension, and dyslipidemia. Donor-related factors included sex, age, BMI, presence of fatty change, and living vs. cadaveric donor. Surgery-related factors of interest were blood type compatibility, cold ischemic time, warm ischemic time, graft type, graft weight, and graft-to-recipient body weight ratio. Postoperative data consisted of follow-up duration, types of calcineurin inhibitors, corticosteroid dose, presence of acute rejection, presence of steroid pulse, mycophenolate mofetil use, presence of end-stage renal disease, and transient hemodialysis. A diagnosis of HCC and determination of the underlying pathology necessitating LT were based on the pathologic results from the recipient’s resected liver. Patients taking insulin or oral hypoglycemic agents or with fasting plasma glucose ≥126 mg/dl, confirmed by repeat testing, or HbA1c ≥6.5% during the preoperative period were considered to have DM. Patients taking medications aimed at treating hypertension and dyslipidemia were considered to have the respective abnormalities. Medical charts were also referred to regarding the cause of death of the patients.

### Immunosuppressive Treatment

Basiliximab was used as an induction therapy in immunosuppression. Standard immunosuppressive therapy usually consisted of a calcineurin inhibitor (tacrolimus or cyclosporine), mycophenolate mofetil, and corticosteroid. If acute rejection was suspected and confirmed by liver biopsy, high-dose methylprednisolone was given and then tapered.

### Sarcopenia Assessment

Patients underwent an abdominal CT scan just before and at a mean of 1 year after LT. The cross-sectional areas and mean Hounsfield units of the psoas muscle, which is a core abdominal muscle, were measured at the L4 level (Aquarius Workstation; TeraRecon, San Mateo, CA, USA. Total fat area and mean Hounsfield units were quantified on the same image. The data were normalized for height^2^ and calculated skeletal muscle index as reported elsewhere [16]. Sarcopenia cutoffs were determined by measuring the cross-sectional area of the psoas muscle in healthy individuals because muscle mass differs not only according to age and sex but also ethnicity [10, 21]. The healthy controls had undergone an abdominal CT scan during a health checkup. Their data were reviewed for medical history and medication use that could affect muscle mass. Sarcopenia was designated if the skeletal muscle index of the psoas muscle was lower than that of the sex- and age-specific 5^th^ percentile [17].

### Statistical Analyses

The data were analyzed using SPSS (version 19.0; SPSS Inc., Chicago, IL, USA) and the R software packages (R version 3.1.2; R Foundation for Statistical Computing, Vienna, Austria; http://www.R-project.org/). Continuous variables are presented as means ± standard deviations. Student’s t-tests were used in comparisons between two groups. Categorical variables are presented as numbers and percentages and were compared using the χ^2^ test with Yates' continuity correction. Kaplan–Meier curves were used to analyze survival, and the log-rank test to compare the curves. A forest plot showed the univariate hazard ratios (HRs) for the survival of post-LT patients. Multivariate-adjusted HRs for the survival of post-LT patients were analyzed using Cox proportional hazard model. Harrell's concordance index was used to assess the prognostic ability of the model in univariate and multivariate Cox regression analyses. A two-sided p-value <0.05 was considered to be statistically significant.

## Results

### Study Population

Table 1 lists the preoperative, operative, and postoperative characteristics of the 145 LT patients. The majority (80%) of the patients were male and the mean age and BMI at the time of LT were 50.2 ± 7.9 years and 24.0 ± 3.1 kg/m^2^, respectively. Mean Child–Pugh and model for end-stage liver disease scores were 7.7 ± 2.6 and 14.4 ± 8.0, respectively. The mean level of fasting glucose was 107.1 ± 34.9 mg/dl; 32 patients (22%) had DM. The most common indication for LT was a hepatitis B virus-associated disease such as hepatitis B virus cirrhosis and/or HCC (84%). The mean age of the donors was 31.4 ± 11.1 years and 80% of the LTs were from living donors. ABO-incompatible LT was performed in 13 cases (9%). The mean cold ischemic time was 171 ± 105 min, and the mean follow-up duration was 51.6 ± 32.9 months. Tacrolimus, as a calcineurin inhibitor, was administered to 76% of the recipients: corticosteroid doses at 3 months and 6 months were 2.3 ± 3.2 mg and 1.0 ± 4.7 mg, respectively. Compared to the post-LT non-sarcopenic group, in the post-LT sarcopenic group, most patients were male (n = 60, 91%, p = 0.003) and the majority had pre-LT sarcopenia (n = 52, 79%, p < 0.001); their mean BMI was also lower (23.3 ± 3.1 kg/m^2^, p = 0.015). Among the 145 patients, 33 recipients (23%) died: 12 in the post-LT non-sarcopenic group and 21 in the post-LT sarcopenic group. Death was most often due to recurrent HCC (n = 19, 58%), followed by de novo cancer (n = 5, 15%), including advanced gastric cancer, colon cancer, and acute myeloid leukemia. Ten patients died due to cancer in the post-LT non-sarcopenic group, and 16 died in the post-LT sarcopenic group. Cancer-specific mortality was not different between the two groups (log rank test, p = 0.116).

**Table 1:** Baseline characteristics of the liver transplantation (LT) recipients.

	Total (n = 145)	Post-LT non-sarcopenia (n = 79)	Post-LT sarcopenia (n = 66)	p-value^a^
**Recipients**				
Male (%)	116 (80)	56 (71)	60 (91)	0.003
Age (years)	50.2 ± 7.9	50.2 ± 7.8	50.1 ± 8.1	0.943
BMI (kg/m^2^)	24.0 ± 3.1	24.6 ± 3.0	23.3 ± 3.1	0.015
Child-Pugh score	7.7 ± 2.6	7.4 ± 2.5	8.2 ± 2.8	0.080
MELD score	14.4 ± 8.0	13.8 ± 8.5	14.9 ± 7.4	0.419
Creatinine (mg/dl)	0.98 ± 0.69	1.05 ± 0.85	0.89 ± 0.40	0.152
Fasting glucose (mg/dl)	107.1 ± 34.9	102 ± 23	113 ± 45	0.096
DM (n, %)	32 (22)	16 (20)	16 (24)	0.688
Pathology necessitating transplantation (HBV-associated, n, %)	122 (84)	71 (90)	51 (77)	0.043
HCC (n, %)	96 (66)	54 (69)	42 (64)	0.599
Hypertension (n, %)	41 (28)	25 (32)	16 (24)	0.359
Dyslipidemia (n, %)	6 (4)	2 (2.5)	4 (6)	0.411
Pre-LT sarcopenia (n, %)	52 (36)	0 (0)	52 (79)	<0.001
**Donors**				
Male (%)	84 (58)	47 (62)	37 (59)	0.730
Age (years)	31.4 ± 11.1	30.6 ± 10.6	32.4 ± 11.6	0.334
BMI (kg/m^2^)	22.9 ± 3.2	23.3 ± 3.8	22.4 ± 2.4	0.077
Presence of fatty change (n, %)	70 (48)	35 (44)	35 (53)	0.320
Living donor (n, %)	116 (80)	66 (84)	50 (76)	0.299
**Surgical factors**				
Blood combination (identical/compatible/incompatible, n, %)	104/28/13 (72/19/9)	55/15/9 (70/18/11)	49/13/4 (74/20/6)	0.533
Cold ischemic time (min)	171 ± 105	161 ± 108	183 ± 100	0.205
Warm ischemic time (min)	44 ± 16	44 ± 14	44 ± 18	0.967
Graft type (right/left/whole, n, %)	98/15/30 (68/10/21)	56/9/13 (72/12/17)	42/6/17 (65/9/26)	0.374
Graft weight (g)	831 ± 431	800 ± 421	868 ± 443	0.346
GRWR (%)	1.2 ± 0.6	1.1 ± 0.6	1.2 ± 0.7	0.353
**Imunosuppressions** ^b^				
Calcineurin inhibitor (tacrolimus/CSA/alteration^c^, n, %)	110/24/11 (76/17/8)	60/11/8 (76/14/10)	50/13/3 (76/20/5)	0.333
Corticosteroids at 6 months (mg/day)	1.0 ± 4.7	0.3 ± 1.6	1.8 ± 6.6	0.075
Steroid pulse for acute rejection (n, %)	35 (24)	16 (20)	19 (29)	0.248
MMF use at 6 months (n, %)	86 (59)	45 (58)	41 (62)	0.613

Continuous variables are presented as means ± standard deviations and were analyzed using Student’s t-tests. Categorical variables are presented as numbers and percentages and were compared using the χ^2^ test with Yates' continuity correction.

BMI, body mass index;

MELD, model for end-stage liver disease;

DM, diabetes mellitus;

HBV, hepatitis B virus,

HCC, hepatocellular carcinoma;

LT, liver transplantation;

GRWR, graft-to-recipient body weight ratio;

CSA, cyclosporine;

MMF, mycopenolate mofetil

^a^Based on a comparison of post-LT non-sarcopenia patients and post-LT sarcopenia patients

^b^Postoperative immunosuppressive treatment

^c^Change in calcineurin inhibitors during the postoperative period

### Changes in Sarcopenic Status during the Peri-Transplant Period

The sex- and age-specific cutoff levels of sarcopenia were 7.7 cm^2^/m^2^ for males aged ≥20 years and ≤50 years, 6.6 cm^2^/m^2^ for males aged >50 years, 4.6 cm^2^/m^2^ for females aged ≥20 years and ≤50 years, and 4.4 cm^2^/m^2^ for females aged >50 years (S1 Table).

**Fig 2:**
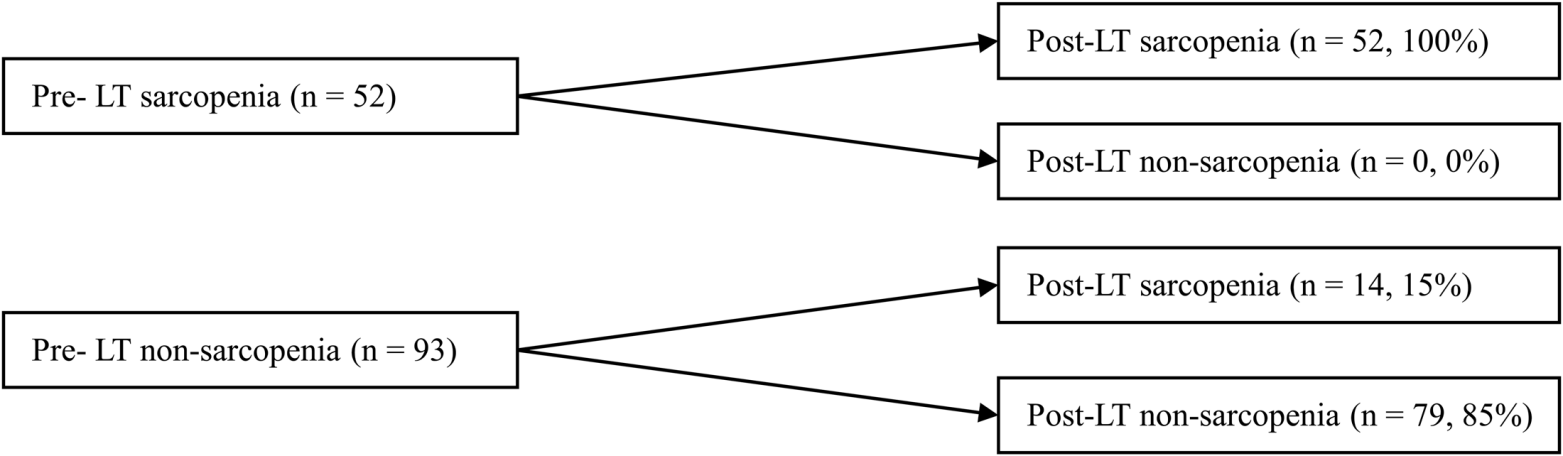
Changes in sarcopenic status during the peri-transplant period. LT, liver transplantation

Preoperative CT scans were performed at a mean of 0.3 ± 0.81 months before LT, and postoperative CT scans at a mean of 12.3 ± 5.7 months after LT. Pre-LT sarcopenia was diagnosed in 52 (36%) patients (Fig 2), with a greater prevalence in males than in females (41% vs. 14%, p = 0.008). In all pre-LT sarcopenic patients, sarcopenia persisted and no change was observed in sarcopenic status after LT. Of the 93 patients who did not have pre-LT sarcopenia, 14 (15%) newly developed sarcopenia after LT. Therefore, the prevalence of post-LT sarcopenia increased to 46% (n = 66) at 1 year after LT.

### Post-LT Survival of Sarcopenic vs. Non-Sarcopenic Patients

**Fig 3:**
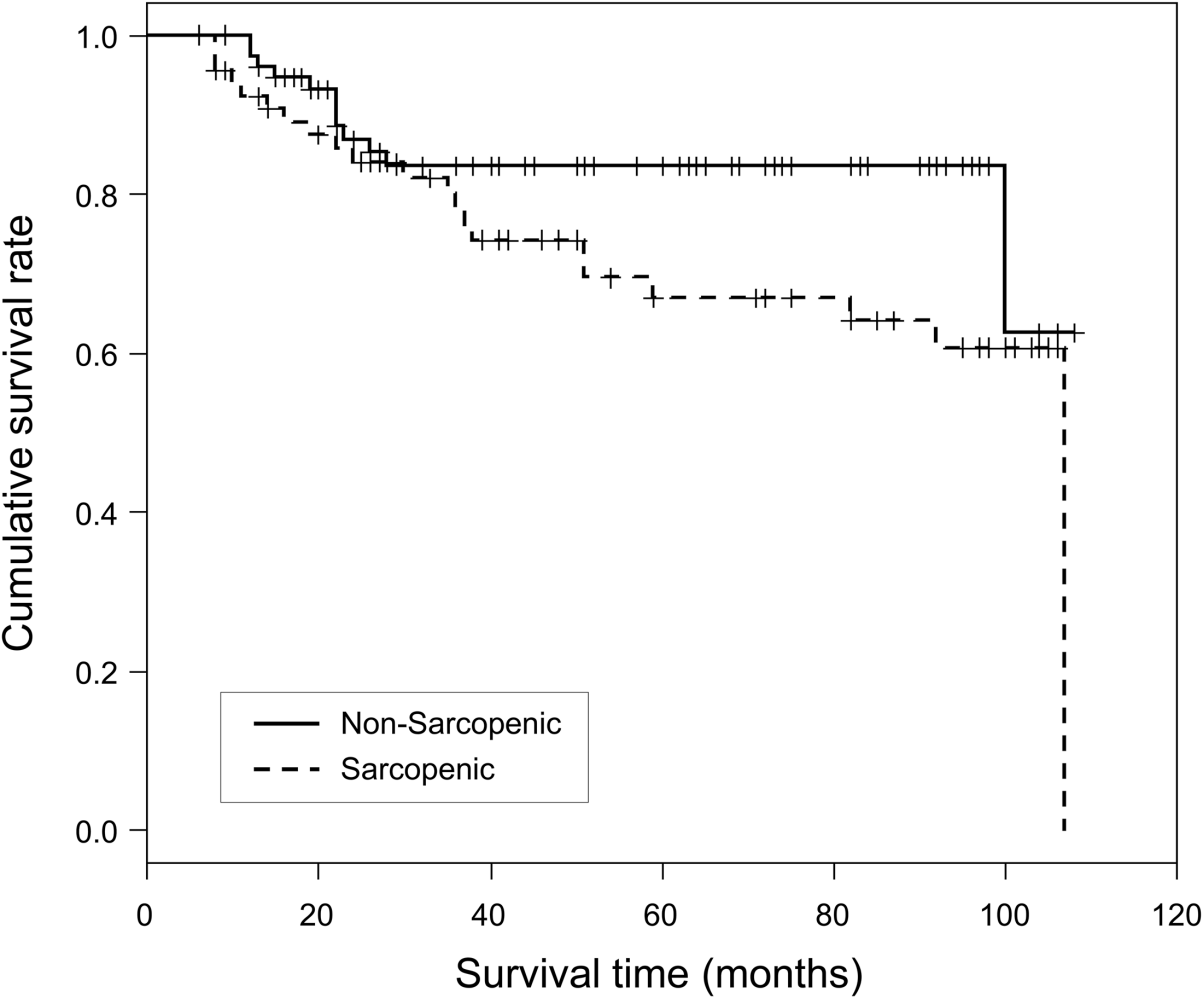
Kaplan–Meier survival curves in post-liver transplantation sarcopenic and non-sarcopenic patients with survival >6 months. Log rank test p-value = 0.069.

The mean survival time was 91.8 ± 4.2 months for non-sarcopenic patients and 80.0 ± 5.2 months for sarcopenic patients; the difference was only marginally significant (log-rank test, p = 0.069; Fig 3). The respective survival rates at 24 and 60 months were 84% and 67% for sarcopenic patients and 87% and 83% for non-sarcopenic patients.

**Fig 4:**
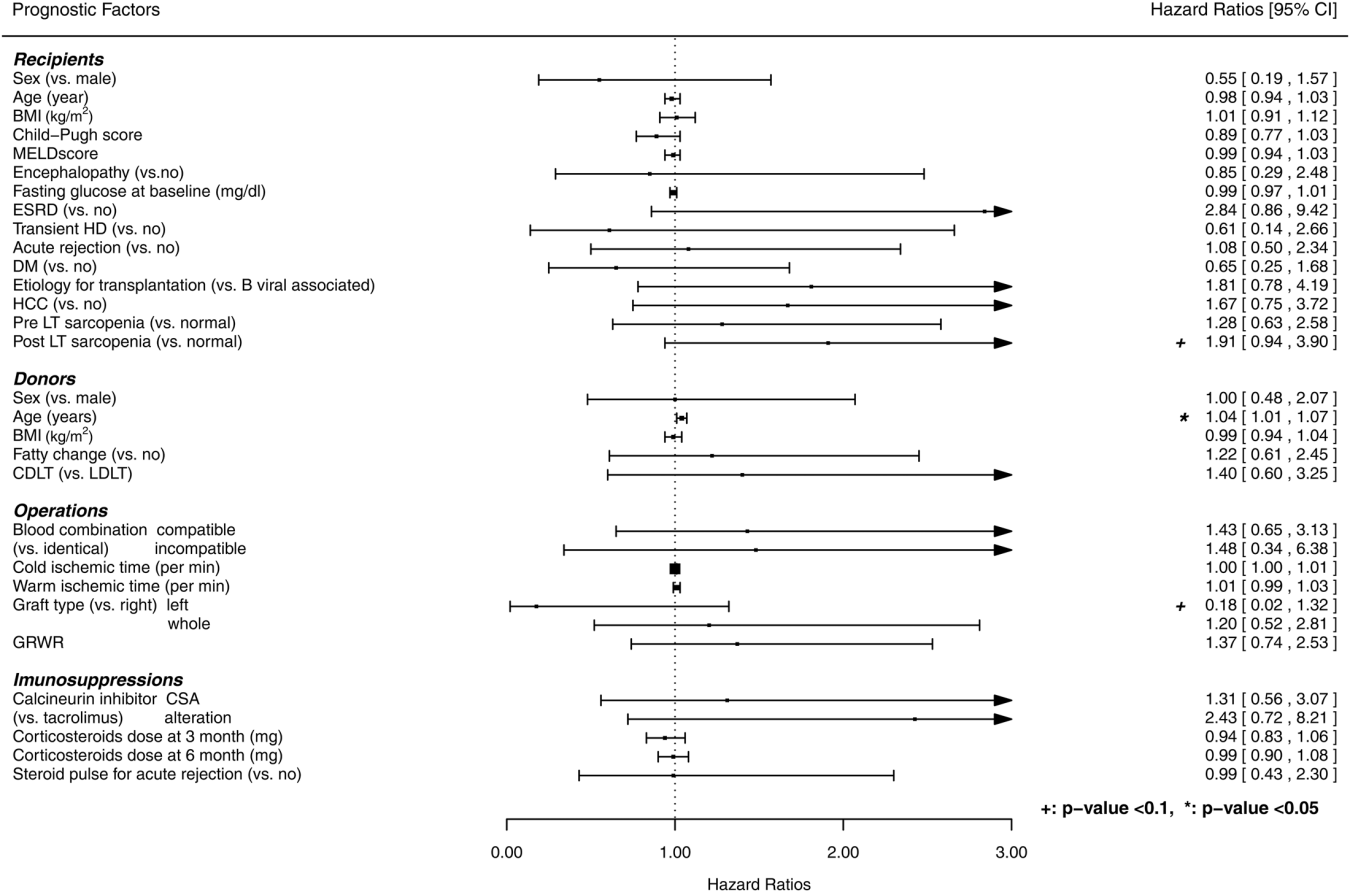
Forest plot of hazard ratios for the associations between post- liver transplantation sarcopenia and survival. BMI, body mass index; MELD, model for end-stage liver disease; ESRD, end-stage renal disease; HD, hemodialysis; DM, diabetes mellitus; HCC, hepatocellular carcinoma; LT, liver transplantation; CDLT, cadaveric donor liver transplantation; LDLT, living donor liver transplantation; GRWR, graft-to-recipient body weight ratio; CSA, cyclosporine; CI, confidence interval

Recipient-, donor-, and operation-related factors and immunosuppression were analyzed for their potential as independent predictors of long-term survival in patients who survived at least 6 months after LT. Univariate analysis revealed older donor age as a significant risk factor for post-LT survival. Post-LT sarcopenia and graft type were of borderline significance for post-LT survival (Fig 4). A multivariate Cox regression model showed that the prognostic ability of Harrell's concordance index model increased (from 0.57 to 0.72), but post-LT sarcopenia was not a significant predictor of post-LT survival (HR: 1.78, 95% confidence interval: 0.74–4.28, p = 0.196; Table 2).

**Table 2:** Multivariate-adjusted hazard ratio of mortality after liver transplantation (LT) for post-LT sarcopenia.

	HR (CI)		HR (CI)	
	Unadjusted	p*-*value	Model 1^a^	p*-*value
Post-LT sarcopenia	1.91 (0.94–3.90)	0.075	1.78 (0.74–4.28)	0.197
Harrell's C (± SE)	0.57 (0.05)		0.72 (0.06)	

LT, liver transplantation;

HR, hazard ratio;

CI, confidence interval;

Harrell's C, Harrell's concordance index

^a^Adjusted factors: age, sex, model for end-stage liver disease score, pathology necessitating transplantation (hepatitis B virus-associated disease), hepatocellular carcinoma, cold ischemic time, diabetes mellitus, steroid dose at 6 months, living or cadaveric donor LT, donor age, donor sex, donor fatty change, graft types

### Post-LT Survival in Patients with Newly Developed Sarcopenia and Sustained Non-Sarcopenia during the Peri-Transplant Period

**Fig 5:**
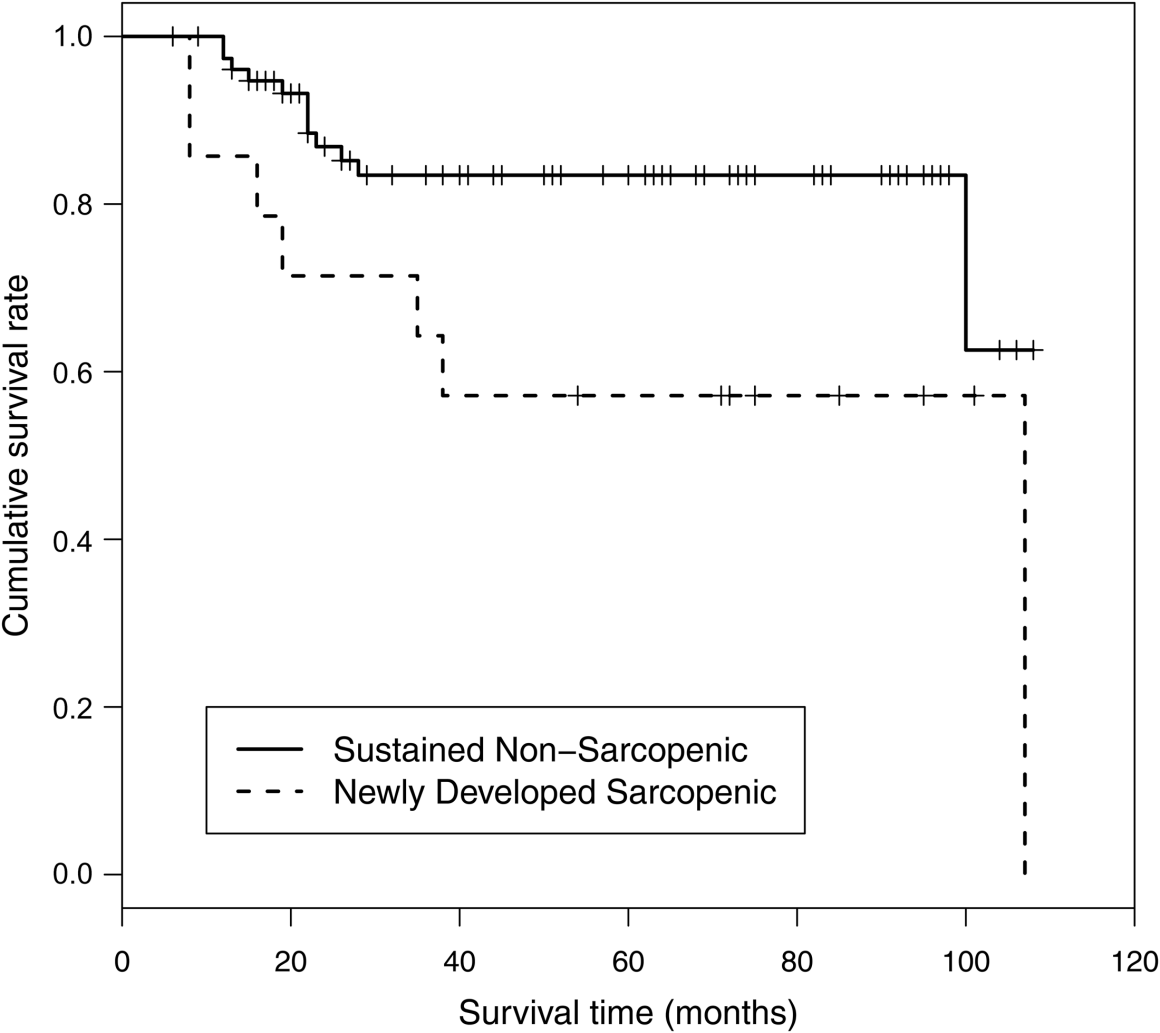
Kaplan–Meier survival curves of patients who remained non-sarcopenic vs. those with newly developed sarcopenia. Log rank test p-value = 0.027.

Survival in recipients who developed sarcopenia after LT was significantly shorter than that of recipients who maintained their non-sarcopenic status after LT (log rank test, p = 0.027; Fig 5). In a multivariate Cox regression model, newly developed sarcopenia after LT (HR: 10.53, 95% confidence interval: 1.37–80.93, p = 0.024) was an independent negative predictor of post-LT survival (Table 3). No significant differences were observed in liver or renal function between the newly developed sarcopenia group and the sustained non-sarcopenic group (S2 Table).

**Table 3:** Multivariate-adjusted hazard ratio of mortality after liver transplantation (LT) for newly developed sarcopenia.

	HR (CI)		HR (CI)	
	Unadjusted	p*-*value	Model 1^a^	p*-*value
Post-LT sarcopenia	2.81 (1.08–7.31)	0.034	10.53 (1.37–80.93)	0.024
Harrell's C (± SE)	0.60 (0.05)		0.81 (0.08)	

LT, liver transplantation;

HR, hazard ratio;

CI, confidence interval;

Harrell's C, Harrell's concordance index

^a^Adjusted factors: age, sex, model for end-stage liver disease score, pathology necessitating transplantation (hepatitis B virus-associated disease), hepatocellular carcinoma, cold ischemic time, diabetes mellitus, steroid dose at 6 months, living or cadaveric donor LT, donor age, donor sex, donor fatty change, graft types

## Discussion

In this study, we showed that the incidence of sarcopenia increased after LT, and that although post-LT sarcopenia was not a predictor for LT survival in patients who survived beyond 6 months after LT, newly developed sarcopenia after LT was associated with increased mortality.

The first few months after LT are a critical period for these patients because the rates of mortality and re-transplantation are high during this time [3]. The effect of sarcopenia on postoperative survival can be masked by many factors, especially biliary complications, bloodstream infection, and a primarily nonfunctioning liver [3]. Therefore, our study population included only those patients who survived 6 months after LT. In this group, we investigated the impact on survival of post-LT rather than pre-LT sarcopenia. In our Kaplan–Meier survival curves, the survival of post-LT sarcopenic and non-sarcopenic patients differed beginning around 30 months after LT, although the difference was only marginally significant according to a univariate analysis and not significant in a multivariate adjusted analysis. Our results are in agreement with those of Tsien et al [17], who showed that a reduction in core muscle mass and the resolution of sarcopenia during LT were of only borderline statistical significance with respect to post-LT survival according to a univariate analysis (p = 0.08) In subgroup analysis, newly developed sarcopenia was a negative predictor of overall post-LT survival and the prognostic ability of the adjustment model (Harrell's concordance index) increased from 0.60 to 0.82. Our data suggest that a change in sarcopenic status during the perioperative period has a greater association than either pre- or post-LT sarcopenia with the survival of LT patients.

Recovery from the metabolic and clinical consequences of cirrhosis is commonly achieved by LT patients after transplantation [22]. However, despite the improved nutritional status, due to restored carbohydrate, lipid, and protein metabolism by the new, functioning liver and to improved dietary intake, sarcopenia may not be reversible, unlike other liver-related complications. In our study, prevalence of pre-LT sarcopenia was 36% (n = 52) and did not show a reversal 1 year after LT; moreover, some patients became newly sarcopenic after LT. Thus, after LT, 66 of the 145 patients (46%) were sarcopenic. Clearly, LT does not guarantee the reversal of sarcopenia, at least 1 year post-LT, as also reported in other studies [17, 23, 24]. The nutritional consequences of patients with end-stage liver disease results in the depletions not only of muscle mass but also of fat mass [23]. Based on a comparison with their preoperative data, the majority of the patients with cirrhosis had a reduction in lean body mass in the immediate postoperative period (10–90 days). The compensatory recovery phase that typically follows LT is more robust for fat tissue than for skeletal muscle and lean body mass based on the current study. In our patients, a comparison of pre-LT and post-LT total fat area with and without normalization to height^2^ showed an increase from 78.5 cm^2^/m^2^ to 83.4 cm^2^/m^2^ and from 220.8 cm^2^ to 235.2 cm^2^, respectively. Several studies have reported that the posttransplant recovery of muscle mass is incomplete until a maximum follow-up of 24 months [24–28]. The pathogenesis of posttransplant sarcopenia is unclear but possible mechanisms include persistent hypermetabolism, the effects of immunosuppressants such as corticosteroids and calcineurin inhibitors, posttransplant infections, renal failure, and relapse of the underlying liver disease [24, 29–31].

Racial differences in body composition, including the amount of muscle and adipose tissue, are well known. In Asians, muscle mass is less than that in whites, blacks, and Hispanics, as determined using dual-energy X-ray absorptiometry [21, 23]. Ours is the first report to determine a sarcopenia cutoff value and to use CT to determine muscle mass in an Asian population, although similar measurements have been made in other ethnic group [17]. Our sarcopenia cutoff was based on measurements of muscle mass in healthy individuals who underwent a CT scan during a health checkup. Consistent with measurements of muscle mass using dual-energy X-ray absorptiometry, our data showed a lower sarcopenia cutoff in Asians than in other ethnicities [10, 17].

The techniques recommended for assessing or estimating muscle mass are DXA, CT, magnetic resonance imaging, and bioimpedance analysis. Of these, CT is considered to be the gold standard based on its higher accuracy and reproducibility in quantifying fat and muscle [4, 32]. The psoas muscle can be easily evaluated on CT because it is surrounded by the vertebra and retroperitoneal fat tissue and is therefore not susceptible to compression by ascites or hepatomegaly. Thus, it allows highly precise measurements of mass for use in quantifying whole-body skeletal muscle volume [33, 34]. LT recipients commonly undergo abdominal CT scanning preoperatively as a part of the routine evaluation and postoperatively for the detection of complications or HCC. Therefore, CT scans in LT recipients are a practical and accessible method of sarcopenia screening and follow-up. In our study, the timing of the CT scans was relatively homogenous compared to other studies and thus yielded reliable data on sarcopenic status before and 1 year after LT.

There are limitations that need to be considered when interpreting the data. First, sarcopenia was diagnosed only on the basis of muscle mass and muscle strength and performance was not considered. However, a reduction of muscle mass is a prerequisite for a diagnosis of sarcopenia. The European Working Group on Sarcopenia in Older People established a staging system of pre-sarcopenia, sarcopenia, and severe sarcopenia based on muscle strength and performance [4]. These parameters, as well as muscle mass, have clinical implications in older individuals and in chronically ill patients [35]. Future studies of the relationship between LT outcome and post-LT sarcopenia should therefore include muscle strength and performance along with muscle mass. Second, the retrospective cohort design did not allow us to draw causal inferences from the observed relationships. Sarcopenia itself has not been demonstrated to be the driver of poor outcomes, although it reflects overall health and has served as a prognostic indicator in patients with cancer and cirrhosis. Therefore it remained unclear whether prevention of post LT sarcopenia through better nutritional and physical therapy programs improves LT outcomes or not. Recently, a small study reported that perioperative nutritional therapy improved survival in patients with sarcopenia [15]. Interventional studies are required to establish whether diet and exercise management after LT reverses sarcopenia in the long term and therefore improves survival. Finally, we only included relatively healthy LT recipients who survived > 6 months, which may have caused selection bias.

## Conclusions

In conclusion, our results show that post-LT sarcopenia was not associated with postoperative survival, whereas postoperative survival was shorter in patients with newly developed sarcopenia after LT. The occurrence of sarcopenia can be used to stratify patients with regard to the risk of post-LT mortality. Thus, we suggest that identifying patients with sarcopenia during the peri-transplant period by quantifying muscle mass should be part of managing these patients.

## Supporting Information

S1 Table Cutoff values for sarcopenia in healthy individuals. Continuous variables are presented as means ± standard deviations (SDs).
(DOCX)

S2 Table Liver and renal function between sustained non-sarcopenia and newly developed sarcopenia after liver transplantation. Continuous variables are presented as means ± standard deviations (SDs). AST, aspartate aminotransferase; ALT, alanine aminotransferase; INR, international normalized ratio.
(DOCX)
