# Supplementary material for: Newly Developed Sarcopenia as a Prognostic Factor for Survival in Patients who Underwent Liver Transplantation
Source: PLoS One. 2015 Nov 30;10(11):e0143966. doi: 10.1371/journal.pone.0143966 (PMC4664264; doi:10.1371/journal.pone.0143966)
Supplement: S1 Table — Continuous variables are presented as means ± standard deviations (SDs). (DOCX) [file pone.0143966.s001.docx]

**S1 Table. Cutoff values for sarcopenia in healthy individuals**

| **Characteristics** | **Males** | | **Females** | |
| --- | --- | --- | --- | --- |
|  | 20–50 years old | >50 years old | 20–50 years old | >50 years old |
| Number | 34 | 29 | 32 | 24 |
| Age (years, mean ± SD) | 29.1 ± 8.4 | 58.2 ± 6.8 | 32.8 ± 7.2 | 58.2 ± 7.2 |
| Psoas muscle |  |  |  |  |
| Absolute (cm^2^) |  |  |  |  |
| 5th percentile | 23.0 | 17.7 | 10.6 | 10.5 |
| Mean | 30.1 ± 8.4 | 27.3 ± 6.1 | 16.9 ± 4.6 | 15.1 ± 2.8 |
| Normalized (cm^2^/m^2^) |  |  |  |  |
| 5th percentile | 7.7 | 6.6 | 4.6 | 4.4 |
| Mean | 10.0 ± 1.3 | 9.4 ± 1.9 | 6.4 ± 1.6 | 6.2 ± 1.1 |
