# Supplementary material for: Newly Developed Sarcopenia as a Prognostic Factor for Survival in Patients who Underwent Liver Transplantation
Source: PLoS One. 2015 Nov 30;10(11):e0143966. doi: 10.1371/journal.pone.0143966 (PMC4664264; doi:10.1371/journal.pone.0143966)
Supplement: S2 Table — Continuous variables are presented as means ± standard deviations (SDs). AST, aspartate aminotransferase; ALT, alanine aminotransferase; INR, international normalized ratio. (DOCX) [file pone.0143966.s002.docx]

**S2 Table.** **Liver and renal function between Sustained non-sarcopenia and newly developed sarcopenia after liver transplantation (LT)**

|  | Sustained non-sarcopenia | Newly developed sarcopenia | *p*-value |
| --- | --- | --- | --- |
| AST (U/L) | 51.2 ± 68.5 | 79.7 ± 102.2 | 0.201 |
| ALT (U/L) | 59.9 ± 108.1 | 104.0 ± 137.2 | 0.181 |
| Albumin (g/dL) | 4.3 ± 0.4 | 4.1 ± 0.5 | 0.158 |
| Bilirubin (mg/dL) | 1.6 ± 2.0 | 3.7 ± 8.6 | 0.399 |
| INR | 1.0 ± 0.1 | 1.0 ± 0.3 | 0.868 |
| Creatinine (mg/dL) | 1.39 ± 1.02 | 1.26 ± 0.51 | 0.664 |
